# False detection and the value of combined tracer mapping in endometrial cancer: a dual case presentation

**DOI:** 10.1093/jscr/rjaf508

**Published:** 2025-08-20

**Authors:** Wiktor Szatkowski, Karolina Pniewska, Paweł Blecharz

**Affiliations:** Department of Gynecological Oncology, Maria Skłodowska-Curie National Research Institute, Garncarska 11, 31-115 Krakow, Poland; Department of Gynecological Oncology, Maria Skłodowska-Curie National Research Institute, Garncarska 11, 31-115 Krakow, Poland; Department of Gynecological Oncology, Maria Skłodowska-Curie National Research Institute, Garncarska 11, 31-115 Krakow, Poland

**Keywords:** endometrial neoplasms, indocyanine green, sentinel lymph node biopsy, technetium Tc99m sulfur colloid

## Abstract

This case report demonstrates two contrasting clinical cases of patients with early-stage endometrial cancer undergoing sentinel lymph node (SLN) mapping. In the first case, a 68-year-old patient with a body mass index (BMI) of 38 underwent SLN mapping using indocyanine green (ICG) alone. Although fluorescence-guided dissection revealed nodes appearing “ICG-positive,” histopathological evaluation confirmed the absence of lymphatic tissue in the removed specimens—indicating the presence of so-called “empty nodes.” This highlights the limitations of relying solely on optical fluorescence, especially in patients with high BMI or altered lymphatic architecture. In contrast, the second case, involving a 65-year-old patient with a BMI of 40, demonstrates the effectiveness of using a combined technique with ICG and technetium-99m (Tc99m). The synergy between real-time fluorescence visualization and gamma probe detection enabled precise SLN identification and confirmed the presence of true lymphatic tissue histologically. This dual case report underscores the limitations of ICG-only SLN mapping and and illustrates the potential added value of technetium-99m in improving the accuracy of lymphatic staging, warranting further validation.

## Introduction

This case report demonstrates two contrasting clinical cases of patients with early-stage endometrial cancer undergoing sentinel lymph node (SLN) mapping. The first patient underwent SLN mapping with indocyanine green (ICG), while in the second case, a dual-tracer approach [(ICG and technetium-99m (Tc99m)] was used. Optimizing SLN mapping in endometrial cancer necessitates careful attention to tracer selection, injection technique, and surgical expertise. While ICG offers real-time visualization advantages, its limitations—such as diffused smearing, swollen lymphatics, and potential false negatives—underscore the need for adjunctive methods. The combined use of ICG and Tc99m enhances detection accuracy, particularly in patients with challenging lymphatic anatomy.

## Case reports

### Case 1

A 68-year-old woman with a BMI of 38 and a diagnosis of endometrioid endometrial adenocarcinoma G1 was referred for surgical treatment. Preoperative imaging, including pelvic magnetic resonance imaging, revealed superficial myometrial invasion without cervical involvement or suspected metastatic spread. Based on these findings, the patient was categorized as low-risk.

**Figure 1:**
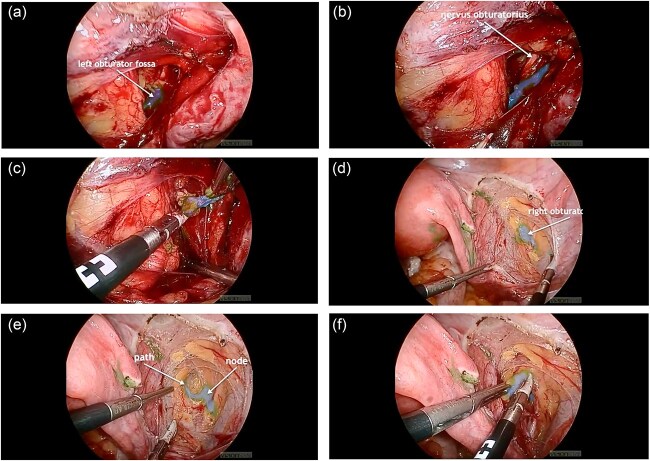
(a) ICG-positive node was visualized in the left obturator fossa; (b) visualization of the left obturator nerve next to the node; (c) node removal on the left side; (d) visualization of the ICG-positive node and the right obturator nerve in the right obturator fossa; (e) the lymphatic path to the node was visualized; (f) node removal on the right side.

The standard procedure involved total hysterectomy with bilateral salpingo-oophorectomy and SLN mapping. Initially, ICG was injected cervically at the 3 and 9 o’clock positions using a combined superficial and deep injection technique. The lymphatic drainage was observed through an intact peritoneum for at least 15 minutes, and the first “ICG-positive” node with a clearly visible afferent lymphatic vessel was identified (Fig. 1).

However, despite the fluorescence signal, histopathological analysis revealed the absence of lymphatic tissue in the excised nodes, indicating removal of “empty nodes.” This finding raised concerns about the reliability of ICG-only mapping in certain anatomical scenarios.

### Case 2

**Figure 2:**
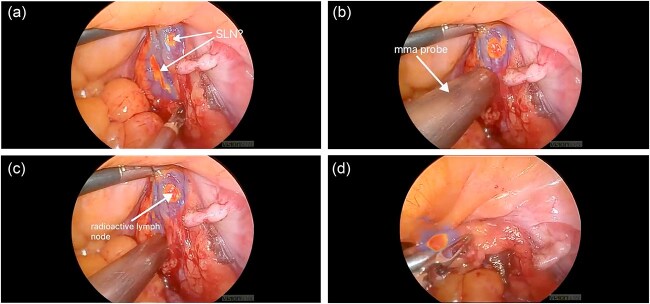
(a) ICG-positive node was visualized; (b, c) gamma probe enabled precise localization of SLN; (d) ICG and Tc99m-positive node was removed.

In a subsequent case—a 65-year-old woman with a BMI of 40 and early-stage endometrial cancer—the SLN procedure was repeated using a combined approach with both ICG and Tc99m. Tc99m was injected into the cervix preoperatively, and single photon emission computed tomography/ computed tomography was used for anatomical mapping. Intraoperatively, a gamma probe enabled precise localization of SLNs, complementing ICG fluorescence (Fig. 2). This approach led to successful bilateral detection of SLNs containing confirmed lymphatic tissue on histopathology.

These two cases illustrate the limitations of ICG-only mapping and demonstrate the enhanced accuracy achieved through a dual-tracer technique, particularly in patients with high BMI and altered lymphatic drainage.

## Discussion

The efficacy of SLN mapping in endometrial cancer remains dependent on both the technical approach and tracer characteristics. A meta-analysis by Xiong *et al.* [1] demonstrated that a lower concentration of ICG (<5 mg/ml) combined with a higher injection volume (≥2 ml) results in improved diagnostic odds ratios, sensitivity, and overall detection rates. These findings emphasize the importance of optimizing injection parameters to ensure consistent lymphatic uptake and effective SLN identification.

However, even when appropriate dosing is used, several technical pitfalls may limit the accuracy of ICG-based mapping. One such issue is diffused smearing, which may result from excessive dye volume, deep or repeated injections, or extensive dissection of retroperitoneal structures. This causes the tracer to leak out of the lymphatic channels, leading to widespread fluorescence and obscuring true SLNs. Another challenge is the misidentification of “swollen lymphatics.” ICG binds to albumin and may draw additional interstitial fluid into the lymphatic vessels due to oncotic pressure, making them appear enlarged and potentially misclassified as lymph nodes during fluorescence imaging [2].

A further limitation arises in cases where no ICG-positive node is visualized, but an afferent fluorescent lymphatic vessel is present. In such scenarios, the downstream non-fluorescent node may still harbor metastasis and is classified as an SLN type 2, highlighting a rare but clinically relevant false-negative phenomenon [3].

To overcome these limitations, the combined use of ICG and Tc99m has been proposed as a more reliable method for SLN mapping. This dual-tracer approach combines the real-time visualization benefits of ICG with the deeper tissue penetration and preoperative imaging capability of Tc99m. Our experience confirms that a dual-tracer approach improves SLN detection accuracy and reduces the likelihood of false-positive intraoperative signals (“empty nodes”) [4]. Importantly, SLN detection rates improve significantly with surgical experience. How *et al.* [5] reported an increase in detection from 77% to 94% after 30 cases, underscoring the value of the learning curve in achieving proficiency with this technique.

In this article, we presented two contrasting cases illustrating the potential limitations of SLN mapping using ICG alone. In our clinical practice, we routinely and successfully apply a combined approach with ICG and Tc99m, which—according to our previous publication [4]—enables more reliable identification of SLNs, especially in patients with challenging anatomical conditions.

## Conclusion

This dual-case video presentation draws attention to potential challenges associated with ICG-only SLN mapping in endometrial cancer, including the risk of “empty node” detection. The use of a combined method with Tc99m may offer practical advantages in certain patients. Although no definitive conclusions can be drawn from this limited series, the cases underscore the value of exploring combined techniques in clinical practice and research.
